# 2D-DIGE proteomic analysis identifies new potential therapeutic targets for adrenocortical carcinoma

**DOI:** 10.18632/oncotarget.3299

**Published:** 2015-01-21

**Authors:** Giada Poli, Elisabetta Ceni, Roberta Armignacco, Tonino Ercolino, Letizia Canu, Gianna Baroni, Gabriella Nesi, Andrea Galli, Massimo Mannelli, Michaela Luconi

**Affiliations:** ^1^ Endocrinology Unit, Department of Experimental and Clinical Biomedical Sciences, University of Florence, Florence, Italy; ^2^ Gastroenterology Unit, Department of Experimental and Clinical Medicine, University of Florence, Florence, Italy; ^3^ Endocrinology Unit, Careggi Hospital, Azienda Ospedaliera Universitaria Careggi, Florence, Italy; ^4^ Division of Pathological Anatomy, Department of Surgery and Translational Medicine, University of Florence, Florence, Italy; ^5^ Istituto Toscano Tumori, Florence, Italy

**Keywords:** proteomics, cancer metabolism, biomarkers

## Abstract

Adrenocortical carcinoma (ACC) is a rare aggressive tumor with poor prognosis when metastatic at diagnosis. The tumor biology is still mostly unclear, justifying the limited specificity and efficacy of the anti-cancer drugs currently available. This study reports the first proteomic analysis of ACC by using two-dimensional-differential-in-gel-electrophoresis (2D-DIGE) to evaluate a differential protein expression profile between adrenocortical carcinoma and normal adrenal. Mass spectrometry, associated with 2D-DIGE analysis of carcinomas and normal adrenals, identified 22 proteins in 27 differentially expressed 2D spots, mostly overexpressed in ACC. Gene ontology analysis revealed that most of the proteins concurs towards a metabolic shift, called the Warburg effect, in adrenocortical cancer. The differential expression was validated by Western blot for Aldehyde-dehydrogenase-6-A1,Transferrin, Fascin-1,Lamin A/C,Adenylate-cyclase-associated-protein-1 and Ferredoxin-reductase. Moreover, immunohistochemistry performed on paraffin-embedded ACC and normal adrenal specimens confirmed marked positive staining for all 6 proteins diffusely expressed by neoplastic cells, compared with normal adrenal cortex.

In conclusion, our preliminary findings reveal a different proteomic profile in adrenocortical carcinoma compared with normal adrenal cortex characterized by overexpression of mainly metabolic enzymes, thus suggesting the Warburg effect also occurs in ACC. These proteins may represent promising novel ACC biomarkers and potential therapeutic targets if validated in larger cohorts of patients.

## INTRODUCTION

Adrenocortical tumors are rather common, with a prevalence of about 9% in the older age, and mostly benign adenomas (ACA). On the other hand, adrenocortical carcinomas (ACC) are rare and aggressive tumors with an incidence of 1-2 cases/million people/year and poor prognosis (5-year survival rate around 30%) [1-4]. Survival rate drops below 10% when metastasis is found at diagnosis.

In spite of recent studies on the genetics and molecular biology of this neoplasia [5-9], its pathogenesis is still not completely understood: new diagnostic and prognostic biomarkers as potential therapeutic targets are sought to improve patient clinical management.

Genetic and molecular studies of the tumor provide information on gene alterations and derangement of the corresponding intracellular pathways. Proteomic studies analyze the physiological or pathological complex of cell proteins, complementing genomic studies which quantitatively and qualitatively examine the end-products of those synthetic cell pathways which may have undergone post-translational modification.

In this paper we present the results of the first 2 dimensional-differential-in-gel electrophoresis (2D-DIGE) proteomic study performed on malignant adrenocortical tumors compared with normal adrenals, in an effort to discover new therapeutic targets for ACC.

## RESULTS

### Differential proteomic profile in ACC versus normal adrenal

The study included 10 patients with histological diagnosis of adrenocortical cancer (ACC), who underwent surgery without any previous anticancer treatment. Clinical characteristics of patients are reported in Table 1.

**Table 1:** Patient characteristics Clinical characteristics of the 10 patients affected by ACC and included in the proteomic analysis. Age at surgery, sex, hormonal activity of the tumor, diameter, Ki67, Weiss score and stage of the tumor, according to the new ACC classification from ENSAT [13] are indicated. For one patient, 2 independent samples were taken in different regions of the tumor biopsy and were analyzed as independent specimens (ACC2 and ACC2a). -: not defined; NS: non secreting; CORT: cortisol, T: testosterone; DHEAS: dehydroepiandrosterone sulfate; DELTA4: androstenedione.

PATIENTS	AGE (years)	SEX	HORMONAL ACTIVITY	DIAMETER (cm)	Ki67 (%)	WEISS	STAGE
ACC1	71	F	NS	9	10	7	4
ACC2 ACC2a	58	F	NS	13	90	8	3
ACC3	36	F	CORT	6.7	15	5	3
ACC4	45	F	DHEAS, T, DELTA4	7.5	5	6	2
ACC11	4	F	-	4	<5	3	-
ACC13	46	F	NS	6	<1	4	2
ACC20	58	F	CORT	7	40	8	3
ACC21	23	F	T, DELTA4	5	1	3	1
ACC22	62	F	NS	2.5	10	6	2
ACC26	1	F	-	9	20	6	-

**Fig. 1:**
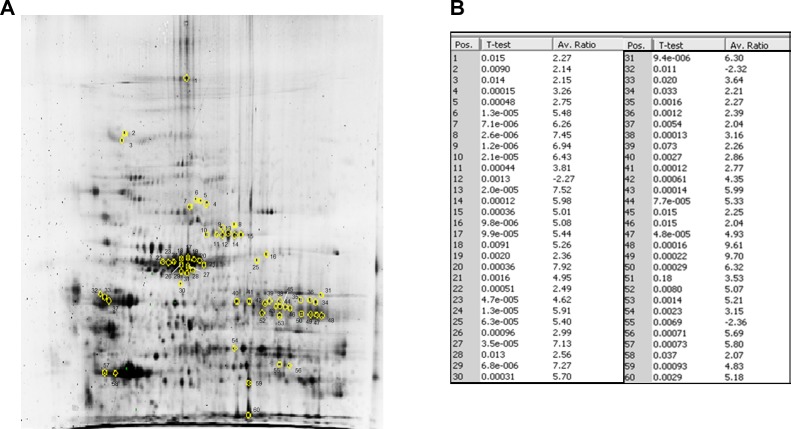
2D-DIGE reveals a profile of 60 protein spots differentially expressed in ACC versus normal adrenal (A) The 60 marked spots in the 2D-DIGE preparative gel correspond to those found differentially expressed between ACC and normal adrenal samples. The spots of interest were picked and subsequently identified by NanoLC-nanoESI-MS/MS and peptide mass fingerprinting analysis as reported in Tab.2. (B) Fold increase expression values for the 60 spots differentially expressed between ACC and NOR as resulted by Decyder Software analysis of the 10 2D-DIGE gels run, reported for each of the 60 marked spots in the preparative gel. Spot identification number (Pos.) as in preparative gel, p-value and average ratio (Av. Ratio) of expression between ACC and normal adrenal samples, evaluated against the internal standard are given for each spot. Only average ratios higher or lower than +2 or −2 were considered. Differences in spot expression between ACC and NOR are considered statistically significant with a p<0.05, Student's *t*-test.

Protein expression profiles for ACC (n=11) and normal adrenal tissue (n=8) lysates obtained by separating samples on 10 parallel 2D gels were compared using 2D-DIGE technology (Fig.1 suppl.). After Decyder analysis of fluorescent gel images, a total of 250 spots were matched across all gels. Gel to gel matching of the standard spot maps from each gel was performed using DeCyder BVA module. This allowed statistical analysis of changes in protein abundance between samples referred to the internal standard. The analysis with BVA module revealed quantitative changes in 60 spots in all samples analyzed, with statistical variance of tumor versus normal spot volume ratios within the 95th confidence level (p<0.05) (Fig.1B). Due to an intrinsic variability associated with patient characteristics and tissue heterogeneity, a stringent criterion was chosen, so that, only proteins found in at least 80% of the 19 tissue specimens (11 ACC and 8 normal adrenals) analyzed in all gels with more than a statistically significant (p<0.05) 2-fold change were considered for further analysis. A total of 60 protein spots were selected and excised from the preparative gel (Fig.1A) for tryptic digestion and mass spectrometry analysis. Analyzed proteins were identified through their peptide spectrum match (PSM) using MASCOT software to interrogate Swiss-Prot database.

Among the 60 spots examined, 27 were finally selected for further analysis. In fact, some of the initially picked spots were discarded as, at variance with those selected, they were not present in 100% of the run gels. Moreover, some of the proteins in the identified 60 spots showed different isoforms or presented post-translational modifications, characterized by different isoelectric point (pI) and molecular weight (MW). When multiple proteins were identified in a single spot, only those exhibiting the number of covering peptides and coverage score over the cut-off values, together with a consistent calculated pI and MW, were considered. Due to the extremely small amount of downregulated proteins between ACC and normal adrenal, most of the detected spots were upregulated. Tab.2 summarizes the characteristics of the spots and correspondent 22 proteins whose expression was modulated with regard to normal tissue extracts.

**Table 2:** List of the 22 differentially expressed proteins between adrenocortical tumors and normal adrenals identified by NanoLC-nanoESI-MS/MS The table shows the MS output list of the 22 proteins identified in the 27 chosen spots that were significantly up- or down-regulated in ACC versus NOR. Fold differences were calculated within the BVA module of DeCyder. All differences are statistically relevant, with p<0.05. Some proteins were present in different spots, generally represented by post-translational modifications. When multiple proteins were identified in a single spot, only those with the number of covering peptides and coverage score over the cut-off values and consistent with the attended pI and MW were considered. The % coverage > 5 or the peptide number ≥ 2 criterion was adopted. Accession number, % coverage, number of covering peptides, peptide spectrum match (PSM), aminoacid number (AA), molecular weight (MW), calculated isoelectric point (calc. pI), score and spot number are indicated.

Accession	Coverage	PSM	# Peptides	# AA	MW [kDa]	pI	Score	Description	Spot
gi4557871	41,26	65	29	698	77,0	7,1	259,38	transferrin [Homo sapiens]	8
gi21614499	37,88	43	23	586	69,4	6,3	146,39	ezrin [Homo sapiens]	9
gi63055049	13,40	15	8	612	68,2	6,7	75,80	phosphoglucomutase 2 [Homo sapiens]	11
gi63055049	10,62	9	6	612	68,2	6,7	45,66	phosphoglucomutase 2 [Homo sapiens]	12
gi4506467	21,78	24	13	583	68,5	6,4	77,36	radixin [Homo sapiens]	13
gi4506467	21,78	24	13	583	68,5	6,4	77,36	radixin [Homo sapiens]	14
gi4506467	21,78	24	13	583	68,5	6,4	77,36	radixin [Homo sapiens]	15
gi66346721	6,56	4	4	640	70,7	7,6	32,12	mitochondrial phosphoenolpyruvate carboxykinase 2 isoform 1 precursor [Homo sapiens]	16
gi5031875	14,69	9	7	572	65,1	6,8	33,97	lamin A/C isoform 2 [Homo sapiens]	30
gi153218646	13,24	11	6	521	60,1	8,8	35,70	cytochrome P450, family 11, subfamily A, polypeptide 1 isoform a precursor [Homo sapiens]	34
gi20070125	27,95	29	13	508	57,1	4,9	179,85	prolyl 4-hydroxylase, beta subunit precursor [Homo sapiens]	33
gi20070125	29,72	108	16	508	57,1	4,9	518,91	prolyl 4-hydroxylase, beta subunit precursor [Homo sapiens]	32
gi18201905	8,60	6	3	558	63,1	8,3	40,59	glucose phosphate isomerase [Homo sapiens]	36
gi195972866	5,11	4	2	584	58,8	5,2	11,84	keratin 10 [Homo sapiens]	37
gi4885281	19,00	14	9	558	61,4	7,8	79,49	glutamate dehydrogenase 1 [Homo sapiens]	39
gi4507115	9,13	7	4	493	54,5	7,2	32,94	fascin 1 [Homo sapiens]	41
gi91199540	14,54	15	6	509	54,1	7,9	79,58	dihydrolipoamide dehydrogenase precursor [Homo sapiens]	42
gi50301238	10,73	8	4	522	56,2	8,5	35,93	glutathione reductase [Homo sapiens]	44
gi50301238	17,05	14	7	522	56,2	8,5	59,59	glutathione reductase [Homo sapiens]	45
gi50301238	17,05	14	7	522	56,2	8,5	59,59	glutathione reductase [Homo sapiens]	46
gi11095441	8,79	8	5	535	57,8	8,5	37,21	aldehyde dehydrogenase 6A1 precursor [Homo sapiens]	47
gi4757810	12,84	10	6	553	59,7	9,1	40,87	ATP synthase, H+ transporting, mitochondrial F1 complex, alpha subunit precursor [Homo sapiens]	48
gi4757810	12,84	10	6	553	59,7	9,1	40,87	ATP synthase, H+ transporting, mitochondrial F1 complex, alpha subunit precursor [Homo sapiens]	49
gi111118981	13,44	12	6	491	53,8	8,4	38,38	ferredoxin reductase isoform 1 precursor [Homo sapiens]	50
gi5453595	18,32	10	7	475	51,6	8,02	37,18	adenylyl cyclase-associated protein [Homo sapiens]	53
gi48255966	16,73	17	8	508	56,9	8,1	74,66	UDP-glucose pyrophosphorylase 2 isoform a [Homo sapiens]	53
gi4758958	10,15	10	3	404	45,5	5,0	48,63	cAMP-dependent protein kinase, regulatory subunit alpha 2 [Homo sapiens]	58
gi17402865	28,28	14	7	297	33,4	7,3	74,87	thiosulfate sulfurtransferase [Homo sapiens]	60

### Hierarchical clustering

**Fig. 2:**
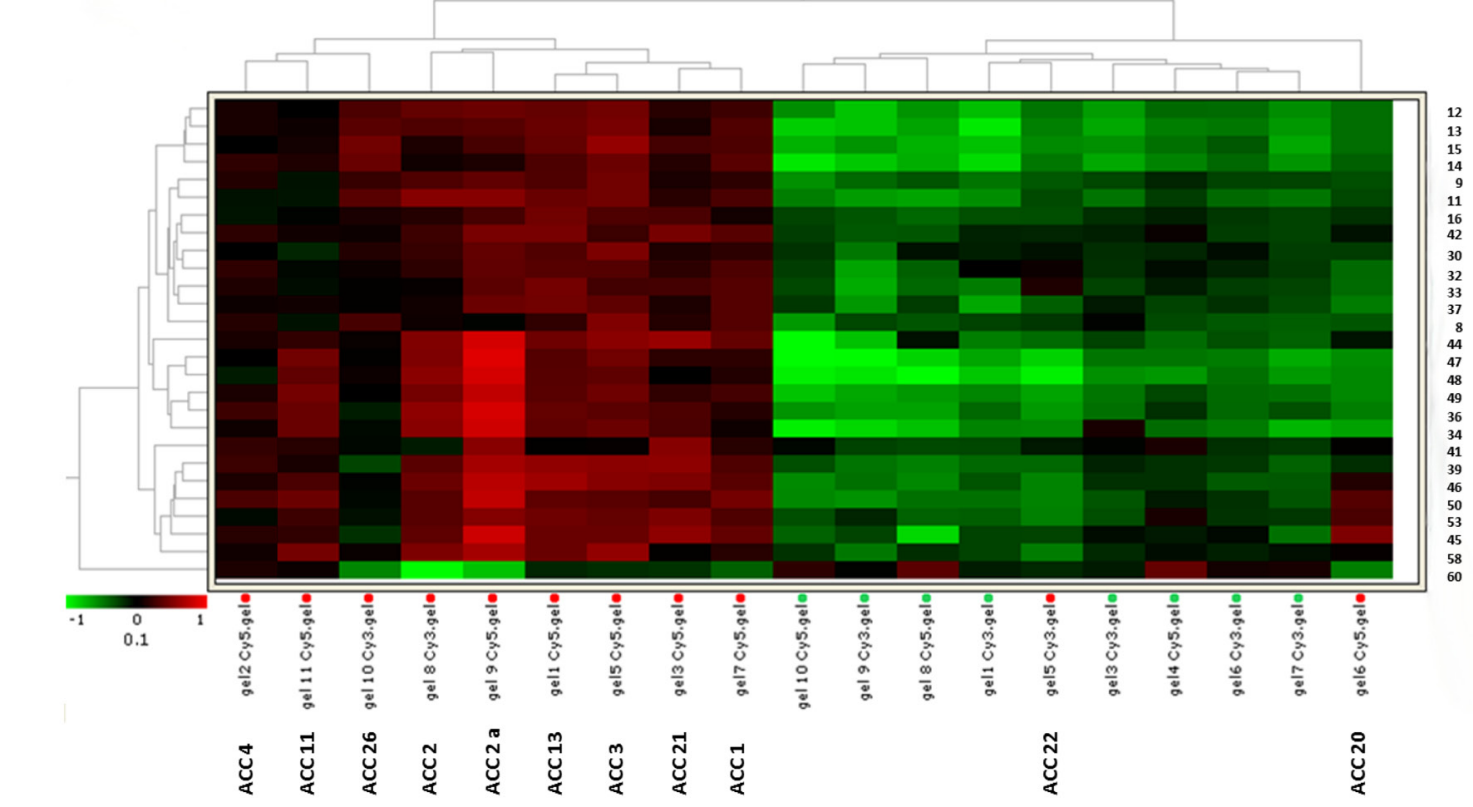
Heat-map for the 27 selected spots identifies two independent clusters Hierarchical analysis of spot expression levels in 19 unilateral adrenocortical tissue specimens from 10 different ACC patients (11 ACC samples) and 8 normal adrenals (NOR). Hierarchical clustering of samples based on 2D-DIGE analysis of the 27 protein spots (right) differentially expressed between ACC and NOR. The heat-map shows expression level of the 27 protein spots in each sample run on 10 2D-DIGE independent gels. Samples and separating gels are indicated at the bottom. The dendrogram shows the degree of similarity of protein expression pattern between tumors and normal tissues. The shorter the branches, the more similar the two joined samples. Two expression patterns of tumor tissue samples resemble more normal adrenals. Heat-map analysis confirmed a similar level of expression in the two independent specimens obtained from ACC2 (ACC2 and ACC2a). The lowest and highest intensity values for each protein are in green and red according to the indicated color scale.

To further investigate the clinical value of the protein expression profile detected in our specimens, we performed with DeCyder a specific hierarchical clustering analysis of the 27 selected spots. Fig.2 shows the heat map obtained from spot fold variation in expression compared with the internal standard in each sample separated on 10 independent 2D-DIGE gels. The expression profile separated samples into two distinct homogeneous clusters corresponding to malignant tumors (overexpression in red) and normal adrenals (downregulation in green), except for 2 out of 11 ACC samples (18%) which were misclassified in the normal adrenal cluster. All normal adrenal samples were correctly classified with no false positive.

### Functional classification of the identified proteins and biological network analysis

**Fig. 3:**
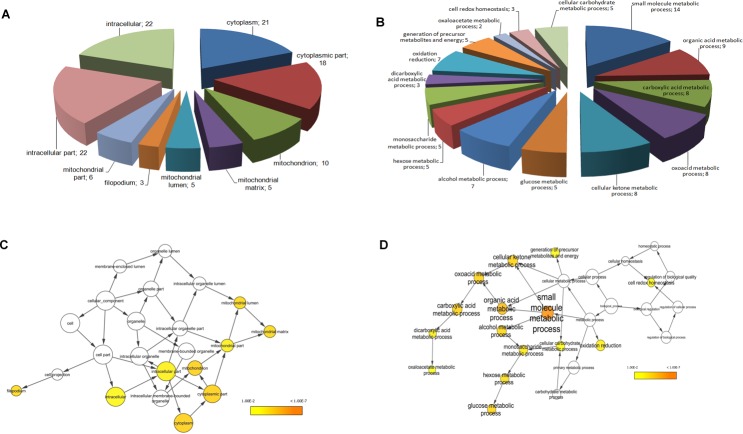
Functional classification of identified proteins and biological network analysis Cellular component (A) and biological processes (B) interested by the 22 differentially expressed proteins assessed by BINGO search and summarized according to functions and location in the cell. For each biological process and cellular component the number of proteins in each class is given. Enrichment analysis of cellular component (C) and biological process networks (D) generated by BINGO software for the 22 differentially expressed proteins. The color bar in right lower quadrant indicates the level of significance from low (yellow) to high (orange). The size of each node is proportional to the number of proteins annotated with that term. Only statistically significant sub-networks are shown in the figure (p<0.01).

Proteins identified by mass spectrometry were classified by subcellular localization and biological processes by BINGO software analysis. As expected, most of the identified proteins were related to the intracellular compartment and more precisely to the cytosol and mitochondria (Fig.3A). Regarding the major biological processes involved, these proteins were mainly associated with induced metabolism (Fig.3B). Enrichment analysis of biological processes obtained through BINGO software (Fig.3D), revealed 15 biological processes enriched in ACC compared with normal controls sorted into the lowest p-value and corrected p-value (Benjamini-Hochberg correction) of 0.01. These processes are mainly involved in metabolism of small molecules, organic acids, ketones, glucose, monosaccharides, alcohols and carbohydrates, in cell redox homeostasis and generation of metabolite precursors and energy (Fig.3D). Instead, the enrichment analysis of cellular compartments showed a prevalent distribution of the 22 proteins differentially expressed at intracellular and mitochondrial level (Fig.3C).

### Validation of differentially expressed proteins by Western blot and immunohistochemical analysis

**Fig. 4:**
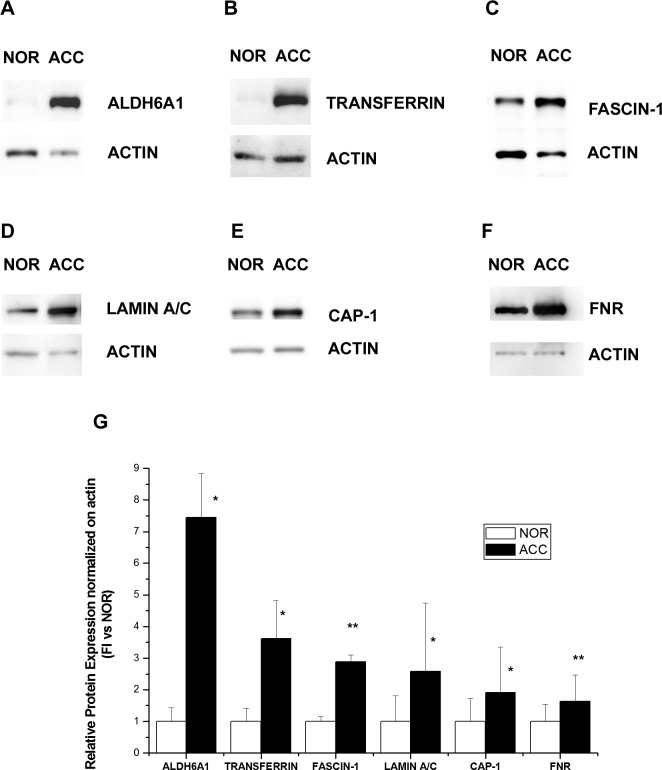
Western blot analysis of six proteins differentially expressed in ACC and normal adrenal Differential expression of ALDH6A1 (A), Transferrin (B), Fascin-1 (C), Lamin A/C (D), CAP-1 (E) and Ferredoxin reductase (FNR, F) as detected by a representative Western blot of the same pool of ACC and normal adrenal (NOR) samples used in 2D-DIGE. (G) Bar charts show mean±SE of relative expression levels for identified proteins vs actin, used as internal loading control and determined by densitometric analysis; n=3 independent Western blots. Statistical analysis with Student's *t* test: *p<0.01, ** p<0.005.

To confirm modulation in the expression of proteins identified by MS after 2D-DIGE analysis, we performed Western blot of the same pooled ACC and normal adrenal specimens. We decided to confirm a set of proteins from the 22 analyzed because of the central role they play in some of the pivotal signaling pathways involved in tumorigenesis and cancer progression. Western blot analysis of lysates containing either all ACC samples (ACC) or all normal adrenal samples (NOR), confirmed the differential expression of Aldehyde-dehydrogenase-6-A1 (ALDH6A1, A), Transferrin (B), Fascin-1 (C), Lamin A/C (D), Adenylate-cyclase-associated-protein-1 (CAP-1, E) and Ferredoxin reductase (FNR, F) in ACC vs. normal adrenals as observed from 2D-DIGE analysis (Fig.4). In particular, in ACC, all proteins were significantly upregulated compared with normal adrenal (Fig.4G), in agreement with the expression ratio observed in 2D-DIGE analysis.

**Fig. 5:**
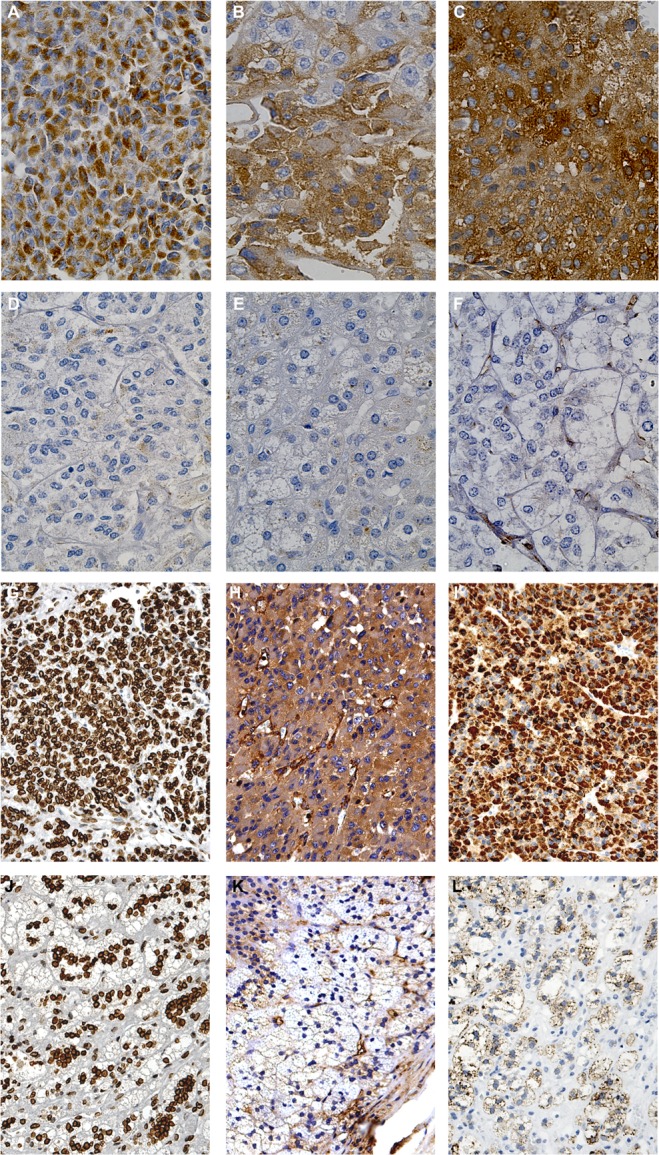
Immunohistochemical detection of the six proteins differentially expressed in ACC and normal adrenal Representative images of immunohistochemistry performed on tumor specimens (n=4) and normal adrenals (n=3) revealed marked positivity to ALDH6A1 (A), Transferrin (B), Fascin-1 (C), CAP-1 (H) and Ferredoxin reductase (I) in the cytosol of almost all tumor cells in the field, compared to no positivity in normal adrenal, respectively (D-F, K-L). Nuclear positivity to Lamin A/C was diffused in tumor cells (G) and less expressed in normal adrenal cortex (J).

To further and independently validate the differentially expressed proteins and localize them into the adrenal tissue, we performed immunohistochemistry for all six proteins on the same ACC and normal adrenal samples (Fig.5). A marked positive granular signal for ALDH6A1 was evident in the cytoplasm of almost all tumor cells, probably associated with mitochondria (Fig.5A), while a more diffuse cytosolic positivity was detected for Transferrin (Fig.5B) and Fascin-1 (Fig.5C) in ACC slices. Marked positivity to Lamin A/C (Fig.5G) was evident in ACC in nuclei and to CAP-1 (Fig.5H) and FNR in cytosol (Fig.5I). Conversely, immunohistochemistry was negative for all 6 markers in normal adrenals (Fig.5D, E, F, K, L), except for a mild positivity to Lamin A/C (Fig.5J), nevertheless lower than the corresponding ACC (Fig.5G).

## DISCUSSION

Differential display analysis of cancer tissues compared with normal counterparts enables definition of specific proteomic profiles characterizing the pathology. Our study, for the first time, applies a 2D-DIGE approach to the analysis of ACC compared with normal adrenal, resulting in identification of a protein pattern specifically overexpressed in ACC. Until now, mostly only transcriptome analysis had been performed on ACC [5-9]. Despite permitting comprehensive analysis of mRNA transcripts, these studies cannot demonstrate that the observed mRNA modulation corresponds to a consequent modulation of the encoded proteins. Indeed, steady-state transcript abundance only partially predicts protein levels in various systems [10]. Immunohistochemistry associated with this approach is limited to the expression analysis of already identified proteins. Moreover, apart from tissue microarray analysis (TMA), not yet been applied to ACC, a simultaneous analysis of the protein complexes likely involved in mediating cancer progression can only be obtained through a proteomic approach. So far, only 2 proteomic studies have been performed in adrenocortical cancer [11]. However, neither study applied 2D-DIGE, but rather narrow range isoelectric focusing (IEF) associated with reversed phase liquid chromatography [11] or conventional 2D electrophoresis [12], failing to directly compare normal and tumor samples. Moreover, classification of tumor and patient characteristics was not conducted according to the accepted ENSAT criteria [13]. Conversely, the 2D-DIGE technique enables direct comparison between tumor and normal samples on the same 2D gel, thus reducing technical variability which could affect any detected differences. Moreover, the introduction of fluorescent dyes to mark the samples to be compared, exponentially increases the sensitivity of detection. Finally, the use of an internal standard, consisting of a mixture of equal amounts of all the samples to be analyzed, allows precise quantification of protein differential expression regardless of the number of matched gels and arbitrary matching of normal and tumor samples in each gel.

The proteomic approach has been widely used to identify useful biomarkers for early detection of various tumors (prostate, breast, colon). In particular, this method has had a relevant clinical impact when biomarkers identified in the tumor were also found to be differentially expressed in the bloodstream [14].

In our study, we compared the proteomic profile of ACC specimens from 10 affected patients with that from normal adrenals. We identified 22 proteins in 27 spots differentially expressed in the pathologic versus normal condition. These proteins can be considered promising biomarker candidates in tumor development and progression. Hierarchical classification of specimens, based on their protein expression profiles with no *a priori* assumption, was associated with the histopathology-based diagnosis of ACC [10], indicating that the proteome profile may well reflect the histological features of the tissue.

In agreement with Kjellin and colleagues [11], who however limited their analysis to the microsomal enriched protein fraction, gene ontology analysis of the 22 differentially expressed proteins found in our study highlighted significant changes in the glucose metabolic pathways in ACC; this strongly indicates a metabolic shift from aerobic respiration to aerobic glycolysis also in adrenocortical tumors. This metabolic reprogramming, also known as the Warburg effect, is a common aspect in various cancer cells that rely on less efficient aerobic glycolysis to achieve their energy requirements, due to defects in the oxidative phosphorylation metabolic pathway [15]. Generation of ATP through glycolysis may better meet the energy needs of rapidly proliferating cells, enabling them to generate the necessary increasing levels of biosynthetic intermediates [16]. In addition, the consequent lactic acid production under aerobic condition may contribute to the oncogenic process via decreased extracellular pH, leading to increased activity of proinvasion factors, such as matrix metalloproteases [17]. Our proteomic analysis identified enzymes acting at the energy-producing metabolic pathway levels that lead to an unusually high production of cancer-promoting compounds. These metabolic and mitochondrial alterations occurring in ACC can offer a window of opportunity for efficient and selective anti-cancer therapies [18], which are still limited for this tumor.

For instance, an increased use of the pentose phosphate pathway may be triggered by the tumor-specific pyruvate kinase isoform (PKM2), which converts phosphoenolpyruvate to pyruvate and is highly expressed in ACC. This enzyme isoform, shifting between a less active dimeric and the tetrameric form, catalyzes the final limiting step in glycolysis, allowing accumulation of phosphometabolites, and so providing a high throughput of supply materials from glucose [19]. Because catalytic activity of PKM2 is lower, it is thought to build up glycolytic intermediates that can be used for anabolic pathways. We can assume that activation of PKM2 depletes intermediates, thus resulting in a reduced proliferation rate of cancer cells.

Aldo-keto reductase 1 member B1 (AKR1B1) that drives glucose flux through the polyol pathway, is also highly expressed in ACC and may play a critical role in tumor development and progression through carbonyl detoxification, retinoic acid homeostatic regulation, and lipid metabolic control [20]. This enzyme is involved in diabetic complications, and a variety of AKR1B1 inhibitors have been developed for the treatment of diabetic patients [21]. The clinical and preclinical data collected over decades by diabetologists would greatly assist the transition of these inhibitors to the clinic of ACC.

One of the most interesting proteins highlighted by 2D-DIGE analysis is ALDH6A1, a member of the aldehyde dehydrogenase family. These enzymes show an increased activity in cancer cells, where they are focused in protecting against apotosis induced by the high levels of reactive oxygen species (ROS) generated in the tumor environment. Nevertheless, this specific isoform is a mitochondrial methylmalonate semialdehyde dehydrogenase that catalyzes valine and pyrimidine catabolism to produce acetyl-CoA, the key hub of metabolic networks. Its upregulation is inversely correlated to progression-free survival in metastatic colon cancer [22]. The lack of the enzyme in normal adrenal versus its high level of diffuse expression in ACC, as further validated by Western blot and immunohistochemical analysis, suggests it could be a promising specific biomarker for future ACC therapies.

A further correlation between glucose homeostasis, lipid metabolism and cell invasiveness in ACC is the expression of adenylyl cyclase-associated protein 1 (CAP-1), involved in the cytoskeletal organization and cell adhesion through its role in activating focal adhesion kinase (FAK) [23]. This protein has recently been demonstrated to be a functional receptor for human resistin, coordinating resistin-mediated inflammation in obesity [24]. Indeed, the adipose tissue surrounding and abundantly infiltrating the adrenocortex might not only act as a passive bystander in ACC. A strict crosstalk between the tumor microenvironment, including the adipocyte components, has recently been claimed to sustain cancer progression, mainly by modulating metabolic activity and providing fuel to cancer cells [25]. A close relationship between obesity and cancer has been suggested, and resistin, which is a specific inflammatory cytokine produced by the adipose tissue, may contribute to supporting the oxidative stress process which regulates tumor metabolic homeostasis [26]. Interestingly, in ACC, CAP-1 expression is accompanied by a parallel upregulation of fascin-1, another actin-bundling protein that crosslinks actin filaments into tight, parallel bundles in filopodia and invadopodia. In pancreatic cancer, a hypoxic tumor microenvironment might promote invasion and metastasis by inducing fascin overexpression. Fascin-1 might be targeted to block cancer progression [27] considering that several small-molecule inhibitors have recently been designed [28].

Contrary to a previous study describing decreased levels of enzymes in the respiratory chain in malignant adrenal tissue [11], our 2D-DIGE analysis could detect only one downregulated protein in tumor condition: thiosulfate sulfurtransferase (TST). Reduced activity of this sulfide-detoxifying enzyme had previously been associated with colon cancer progression [29].

Our study has some limitations, such as the low number of the ACC samples, explained by the rarity of the tumor. In fact, this limited number does not allow any correlation to the patient clinical data. Larger confirmatory multicenter studies are necessary to endorse our results. Moreover, the small number of samples did not allow any specific evaluation of the proteins found as putative biomarkers of ACC by the use of a dedicated software such as EDA. The 2D-DIGE approach may underestimate protein downregulation in ACC compared with normal adrenal. Differences in proteins identified as differentially expressed in our study compared with previous ones [11,12] may be due to the techniques used for protein separation, as well as to differences in tumor samples and corresponding controls (adenoma or normal adrenals). Finally, it was not possible to compare ACC with normal adrenal specimens from the same patients, as the tumor usually invades all normal adrenal cortex, making it difficult to dissect normal tissue. For this reason, we had to use normal adrenals from different donors as controls, thus introducing a further grade of variability.

In conclusion, through the use of a 2D-DIGE approach applied for the first time to the screening of proteomic expression in ACC, we identified an overexpression protein profile associated with ACC. This specific profile suggests metabolic reprogramming from aerobic respiration to aerobic glycolysis, known as the Warburg effect, which has now been suggested to also occur in adrenocortical cancer. The discovery that these key enzymes also play a role in the development and progression of this tumor opens new avenues for their use as potential targets for more efficacious ACC therapies [30]. Immunohistochemistry and Western blot validation of a restricted panel of 6 proteins designates them as potential biomarkers for supporting diagnosis and prognosis. Further studies including larger cohorts of ACC and benign adrenocortical adenomas are needed to validate our encouraging preliminary results and enable correlations with clinical data, in an attempt to confer any diagnostic and prognostic significance to these biomarkers.

## METHODS

### Patients

All patients, or their parents in the case of pediatric patients, gave their written informed consent to the study. The study includes 10 patients affected by ACC, whose clinical characteristics are detailed in Tab.1. All patients underwent surgical removal of the tumor at our University Hospital. Normal adrenal specimens were obtained at radical nephrectomy for renal carcinoma or from organ donors (n=8). Tumor (ACC) specimens and normal adrenal (NOR) samples were snap frozen and stored at −80°C until protein extraction, or were formalin-fixed/paraffin-embedded for immunohistochemistry. Double sampling was performed in tumor biopsy of patient 2 resulting in 2 independent samples to be analyzed (ACC2 and ACC2a).

The study was approved by the Local Ethical Committee.

### 2D-differential-in-gel-electrophoresis (2D-DIGE)

Tissue samples were homogenized by mechanical disruption with Ultraturrax T10 basic IKA (Werke Gmbh & Co, Staufen, Germany) in lysis buffer (30 mM Tris, pH 8.5, 7 M urea, 2 M thiourea, 4% CHAPS). The protein extracts were further treated with the PlusOne 2-D Clean-Up Kit and quantified with 2-D quant Kit (GE Healthcare, Milan, Italy). Fifty μg of each sample were minimally labeled with 400 pmol CyDyes DIGE Fluors (GE Healthcare): Cy3 and Cy5 for ACC (n=11) and NOR (n=8) samples, respectively, and incubated on ice in the dark for 30 min. An internal standard was generated by combining equal amounts of extracts from all ACC and NOR samples and labeled with Cy2.

A total of 10 2D-gels were run separating 2 arbitrarily paired samples of ACC and normal adrenal. The samples were focused using immobilized pH gradient IPG strips (3-10 NL range, 18 cm) onto an IPGphor (GE Healthcare) apparatus (67 kVh, 20°C). After focusing, each strip was equilibrated twice, and then loaded and separated on a 12 % polyacrylamide SDS-PAGE gel using a DALT six (GE Healthcare) apparatus. Images were acquired with a Typhoon TRIO scanner (GE Healthcare) using specific emission filters. Images were analyzed using DeCyder Differential Analysis Software v7.2. Intragel spot detection and quantification were performed by Differential In-gel Analysis (DIA) DeCyder module, while Biological Variation Analysis (BVA) module, which takes into account intergel variability, was used for matching and quantifying the total set of protein spots from all gels simultaneously analyzed. DIA component draws boundaries around spots in a composite gel image obtained from the intragel overlap of the Cy2-, Cy3-, and Cy5-scanned images and normalizes the data from each CyDye to account for differences in dye fluorescence intensity and scanner sensitivity. For all sets of experiments (10 images), the difference in abundance between samples (ACC and NOR) run on the same gel was analyzed. BVA component was then used to match all image comparisons from individual Cy3/Cy5 gel sets for cross-gel statistical analysis. BVA calculates normalized intensities (standard abundance) for all spots by comparison with the internal standard, and then, an average volume ratio and relative p-value by Student's paired *t*-test. Only spots with 2-fold changes in volume after normalization in at least three separate experiments (p<0.05) were considered altered and selected for further characterization.

Extended Data Analysis (EDA) component of DeCyder software was used to construct the heat-map of differential protein expression between tumor and normal adrenal samples.

### Protein identification by NanoLC-nanoESI-MS/MS

Preparative 2D-sodium-dodecyl-sulphate-gel-electrophoresis was run and stained with SYPRO Ruby ((BIO-RAD Labs, Segrate, Milan, Italy) for protein visualization. The spots of interest were automatically detected and excised using an Ettan-Picker robot (GE Healthcare) and subjected to tryptic digestion.

Each peptide mixture was submitted to NanoLC-nanoESI-MS/MS analysis on an Ultimate 3000 HPLC (Dionex, San Donato Milanese, Italy) coupled to a LTQ Orbitrap mass spectrometer (Thermo Fisher, Bremen, Germany). Peptides were concentrated on a precolumn cartridge PepMap100 C18 (300μm i.d.×5mm, 5μm, 100Å, LC Packings Dionex) and then eluted on an Acclaim PepMap100 nano-column (75μm i.d.×15cm, C18, 3μm, 100Å, LC Packings Dionex) at 300 nl/min. The loading mobile phases were: 0.1% TFA in H_2_O (phase A) and 0.1% TFA in CH_3_CN (phase B). The elution mobile phases composition was: H_2_O 0.1% formic acid/CH_3_CN 97/3 (phase A) and CH_3_CN 0.1% formic acid/H_2_O 97/3 (phase B). The elution program lasted: 0 min, 4% B; 10 min, 40% B; 30 min, 65% B; 35 min, 65% B; 36 min, 90% B; 40 min, 90% B; 41 min, 4%B; 60 min, 4% B. Mass spectra were acquired in positive ion mode, setting the spray voltage at 2 kV, the capillary voltage and temperature respectively at 45 V and 200 °C, and the tube lens at 130 V. Data were acquired in data-dependent mode with dynamic exclusion enabled (repeat count 2, repeat duration 15 s, exclusion duration 30 s); survey MS scans were recorded in the Orbitrap analyzer in the mass range 300-2000 m/z at a 15,000 nominal resolution at m/z = 400; then up to three most intense ions in each full MS scan were fragmented (isolation width 3 m/z, normalized collision energy 30) and analyzed in the IT analyzer. Monocharged ions did not trigger MS/MS experiments. The acquired data sought with Proteome Discoverer 1.2 (Thermo Scientific, Waltham, MA, US) using Sequest as search algorithm against Human Protein database. Searches were performed allowing: (i) up to two missed cleavage sites, (ii) 10 ppm of tolerance for the monoisotopic precursor ion and 0.8 mass unit for monoisotopic fragment ions, (iii) carbamidomethylation of cysteine and oxidation of methionine as variable modifications. We only accepted peptides displaying high confidence and proteins with: (i) at least two spectra representing two distinct peptides and (ii) protein score higher than 30. The obtained peptide masses were matched with the theoretical peptide masses of all proteins from the human database of the NCBInr using MASCOT with the automated MASCOT Daemon (Matrix Sciences v2.1, UK).

### Monodimensional sodium-dodecyl-sulphate-gel-electrophoresis (SDS-PAGE) and Western blot analysis

Fifty μg of samples containing equal amounts of all ACC or NOR samples, used for 2D-DIGE, were separated by reducing monodimensional SDS-PAGE (10% polyacrylamide) and transferred to PVDF membranes (Merck-Millipore, Milan, Italy). Protein bands were revealed by primary antibodies (sc-271582 anti-ALDH6A1, sc-52256 anti-Transferrin, sc-46675 anti-Fascin-1, sc-6215 anti-Lamin A/C, sc-376512 anti-CAP-1, sc-374436 anti-Ferredoxin-reductase, sc-1615 anti-Actin, Santa Cruz Biotechnology) followed by peroxidase-secondary IgG and ECL detection kit (Immobilon, Merck-Millipore, Milan, Italy). Image acquisition and relative quantification of band intensity on actin was performed on ChemiDoc XRS instrument with Quantity One software (BIO-RAD Labs, Milan, Italy).

### Functional classification of proteins and pathway analysis

A functional study with Gene Ontology (GO) [31] over-representation analysis was performed with the Biological Network Gene Ontology (BINGO) plug-in for the Cytoscape visualization software [32] correcting p-values for multiple testing and using a Benjamini and Hochberg False Discovery Rate corrected p-value threshold of 0.01 [33].

### Histologic diagnosis and immunohistochemistry

Histologic diagnosis of ACC was performed by reference pathologist on tumor tissue removed at surgery. Tumor specimens were evaluated according to the Weiss System where the presence of three or more criteria highly correlates with malignant behavior [34].

Ki67 index was evaluated as proliferation marker to assess ACC prognosis using anti-human Ki67 monoclonal MIB1 antibody (Dako, Carpenteria, CA, US). Ki67 positive nuclei were counted on 1.000 tumor cells and Ki67 was expressed as the percentage of proliferating cells.

Tumor stage was evaluated according to the revised TNM classification of ACC proposed by the European Network for the Study of Adrenal Tumors [13].

For immunohistochemical analysis, section of 3μm were deparaffinized, hydrated with grade ethanol concentrations until distilled water. Serial sections of the same specimen were immunostained with mouse monoclonal anti-ALDH6A1, anti-Fascin-1, anti-CAP-1, anti-Lamin A/C, anti-Ferredoxin-reductase, anti-Transferrin (dilution 1:50) antibodies after treatement with 3.0% hydrogen peroxidase in PBS (Dako Wash Buffer 10x). Immunohistochemical analysis was carried out using DAKO EnVision™ FLEX (Dako, Carpenteria, CA, US).. Negative control was performed with a non-immune serum.

### Statistical analysis

The relative levels of stained protein spots compared with the internal standard spots were analyzed by DeCyder Difference In-gel Analysis (DIA) and DeCyder Biological Variation Analysis (BVA) software modules (GE Healthcare).

Student's *t*-test was used to calculate statistically significant differences between 2 groups in relative abundance of individual protein spots among the groups in 2D-DIGE and in protein band intensity in Western blot analysis. P<0.05 was considered statistically significant.

## SUPPLEMENTARY MATERIAL FIGURE


