# Supplementary material for: 2D-DIGE proteomic analysis identifies new potential therapeutic targets for adrenocortical carcinoma
Source: Oncotarget. 2015 Jan 21;6(8):5695–706. doi: 10.18632/oncotarget.3299 (PMC4467395; doi:10.18632/oncotarget.3299)
Supplement: Supplementary file 1 [file oncotarget-06-5695-s001.pdf]

2D-DIGE proteomic analysis identifies new potential therapeutic targets for adrenocortical carcinoma

Supplementary Material

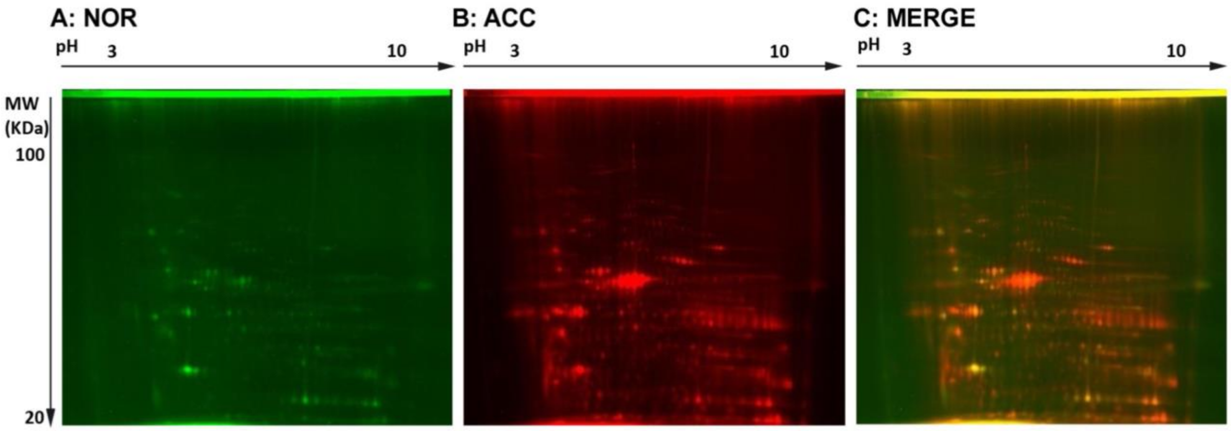

Suppl. Fig.l: Protein Expression Profiling in 2D-DIGE. Representative gel image of proteins differentially expressed in normal adrenal tissue (A, NOR) and ACC (B, ACC). (C) Overlay image of the 2 fluorochromes (MERGE). Proteins extracted from tissue specimens were labeled with the fluorescent dyes Cy3 and Cy5, and separated by 2D-DIGE. lPG strips (3-10 pi) were used for IEF prior to standard SDS-PAGE (12 % polyacrylamide) for the second dimension. Molecular weight (MW) in kilo Daltons (kDa) are indicated to the left of the blots and the pH gradient to the top.
